# Antiviral therapy predicts the outcomes following resection of hepatocellular carcinoma in patients negative for HBV DNA: a propensity score matching analysis

**DOI:** 10.1186/s12957-019-1577-9

**Published:** 2019-03-01

**Authors:** Mingxing Xu, Zheng Zhou, Ruiyun Xu, Huiling Zhang, Nan Lin, Yuesi Zhong

**Affiliations:** 1 Department of Hepatobiliary Surgery, The Third Affiliated Hospital of Sun Yat-Sen University, No. 600 Tianhe Road, Guangzhou, 510630 Guangdong China; 2 Department of Hepatobiliary and Pancreatic Surgery, National Cancer Center/National Clinical Research Center for Cancer/Cancer Hospital and Shenzhen Hospital, Chinese Academy of Medical Sciences and Peking Union Medical College, Shenzhen, China

**Keywords:** Antiviral therapy, Negative HBV DNA, Hepatocellular carcinoma, Survival

## Abstract

**Background:**

The effect of antiviral therapy (AVT) on clinical outcomes in patients with hepatocellular carcinoma (HCC) who are seronegative for hepatitis B virus (HBV), defined as HBV DNA < 100 IU/ml prior to surgical resection, is unknown. The main purpose of this study was to evaluate the possible value of AVT in this cohort of patients.

**Methods:**

From January 2006 to January 2013, 161 HCC patients with positive serum tests for HBV surface antigen (HBsAg) but negative tests for HBV DNA who had undergone hepatectomy were included and analyzed. Propensity score matching (PSM) was used to balance the heterogeneity in baseline characteristics.

**Results:**

All patients were divided into the following two groups: the AVT group (*n* = 73, 45.34%) and the non-AVT group (*n* = 88, 54.66%). HBV reactivation occurred in 20 patients in the non-AVT group (22.73%) but in only 2 patients in the AVT group (2.74%, *p* < 0.001). After PSM, the 1-, 2-, and 3-year recurrence-free survival (RFS) rates in the AVT group and the non-AVT group were 78.38%, 72.97%, and 62.16% and 81.08%, 72.97%, and 72.97%, respectively (*p* = 0.564); the 1-, 2-, and 3-year overall survival (OS) rates were 97.30%, 97.3%, and 91.89% and 94.59%, 94.59%, and 86.49% in the AVT group and non-AVT group, respectively (*p* = 0.447).

**Conclusions:**

Antiviral therapy can reduce HBV reactivation but is not correlated with a significant increase in postoperative RFS and OS in HCC patients with HBV DNA levels < 100 IU/ml.

**Electronic supplementary material:**

The online version of this article (10.1186/s12957-019-1577-9) contains supplementary material, which is available to authorized users.

## Background

With introduction and uptake of the vaccine, hepatitis B virus (HBV) infection rate has not been increasing at the same rate; however, the large population of infected individuals renders the situation less optimistic. Two billion people worldwide have been infected with HBV, the most common pathogen that causes human viral hepatitis, and 250 million are chronically infected [1, 2]. HBV infection is the most common etiological factor leading to hepatocarcinogenesis, disproportionately affecting Asian countries. Globally, the proportion of patients with hepatocellular carcinoma (HCC) caused by HBV infection is 45%, and in China, this proportion has reached approximately 80% [3, 4]. Hepatitis B and its complications, such as cirrhosis or HCC, have contributed to the burden healthcare places on society [5, 6]. Thus, the treatment of chronic hepatitis B patients is particularly important. Antiviral therapy (AVT) is regarded as the most effective treatment. It is expected that AVT can benefit patients with a persistent virological response (VR) [7–9]. Furthermore, AVT can reduce the risk of postoperative recurrence by reducing viral load in HBV-related HCC patients [10]. A recent study also confirmed that AVT is associated with reduced incidences of microvascular invasion (MVI) and early tumor recurrence after partial hepatectomy for HBV-related HCC patients [11]. To improve the prognosis of patients, most major guidelines have recommended AVT as one of the important treatments for HCC patients who test positive for HBV DNA during the perioperative period [12–14]. Previous studies have indicated that AVT can reduce the risk of virus reactivation, help improve liver function in patients with low levels of HBV DNA (HBV DNA < 500 IU/ml) after HCC surgery [15], and reduce HCC recurrence in patients with low HBV DNA levels (HBV DNA < 2000 IU/ml) [16]. However, whether AVT is useful in HCC patients who test negative for HBV DNA (HBV DNA < 100 IU/ml) to improve their clinical outcomes is still unknown, as evidence is lacking.

The present study evaluated the rate of post-hepatectomy HBV reactivation in HBV DNA-negative patients and explored the potential value of AVT for improving patient survival; the hypothesis was that AVT would be beneficial for HBV DNA-negative HCC patients (HBV DNA < 100 IU/ml).

## Materials and methods

All patients in this study provided written informed consent before undergoing operation. All procedures in this study were performed in accordance with the principles of the Research Ethics Committee of the Third Affiliated Hospital of Sun Yat-Sen University and the Helsinki Declaration and its amendments. The study was reported in accordance with the STROCCS criteria [17].

### Study design and patient grouping

We retrospectively collected and analyzed the data of HCC patients who underwent hepatectomy in our institution between January 2006 and January 2013. Patients who met the following inclusion criteria were enrolled in this study: (a) the patient’s hepatectomy was performed in our center, and HCC was pathologically confirmed after the operation; (b) the patient was positive for serum HBsAg before the surgery; (c) the patient’s serum HBV DNA level was < 100 IU/ml; (d) the patient’s liver function was Child-Pugh grade A or B; (e) if the patient received entecavir therapy, it was administered within 1 week before and after the hepatectomy; and (f) if the patient received entecavir (0.5 mg/d), they took it for at least 1 year after surgery.

Patients were excluded if they met one or more of the following exclusion criteria: (a) the patient received treatment that might affect their prognosis before surgery such as transcatheter arterial chemoembolization (TACE) or radiation therapy; (b) the patient had other tumors in other organs, for example, renal cell carcinoma and cervical cancer, or certain severe diseases; (c) the patient had refractory ascites or hepatic encephalopathy; (d) the patient had a positive resection margin (R1 resection); or (e) they had previously received any antiviral treatment.

### Definition of negative HBV DNA, reactivation, PVI, HVI, and MVI

In this study, we defined a negative result for HBV DNA as a serum level of HBV DNA < 100 IU/ml and defined macrovascular invasion as the presence of portal vein invasion (PVI) and/or hepatic vein invasion (HVI), which was detected by two different inspection methods, magnetic resonance imaging (MRI), or computed tomography (CT) accompanied by ultrasonographic testing. All imaging and ultrasonic reports were verified by at least two senior experts in related fields. We defined microscopic vascular invasion (MVI) as the invasion is visible only on microscopy and was assessed by several sections of non-tumoral hepatic parenchyma 1 cm away from the tumor according to previous studies [18, 19]. HBV reactivation was defined as a 10-fold or greater increase in HBV DNA level compared to the baseline level [20, 21].

### Follow-up and outcomes

All patients were followed up once a month in the first 3 months after hepatectomy and then every 3 months in subsequent months until 36 months after surgery or until death. Patients received routine blood tests, liver function tests, serum alpha-fetoprotein (AFP) assays, ultrasound and CT, or MRI at each reexamination. Recurrence-free survival (RFS) and overall survival (OS) were the outcomes of this study.

### Propensity score analysis

Because grouping was based only on perioperative AVT rather than randomization, propensity score matching (PSM) was used to reduce the imbalance between the two groups. All possible clinicopathological covariates, such as tumor number, tumor size, and vascular invasion, which might have affected the target outcomes were included when conducting the PSM. Propensity scores were evaluated using a logistic regression model in this study. Patients were matched at a 1:1 ratio using the caliper matching method within 0.2 of the standard deviation from the propensity score logit, basing on previous literatures [22–24]. And finally, we obtained the score-matched pairs for subsequent analyses.

### Statistics and analysis

Intergroup categorical data were analyzed with the *χ*^2^ test or Fisher’s exact test, as appropriate. Univariate and multivariate analyses were performed using the Cox proportional hazards model. Survival curves were calculated and compared using the Kaplan-Meier method and log-rank test. All analyses were performed using SPSS version 22.0 (IBM, United States), and the PSM analysis was conducted in R (R Foundation for Statistical Computing, Austria). A two-tailed *P* value of less than 0.05 was considered statistically significant.

## Results

### Clinicopathological characteristics

**Fig. 1:**
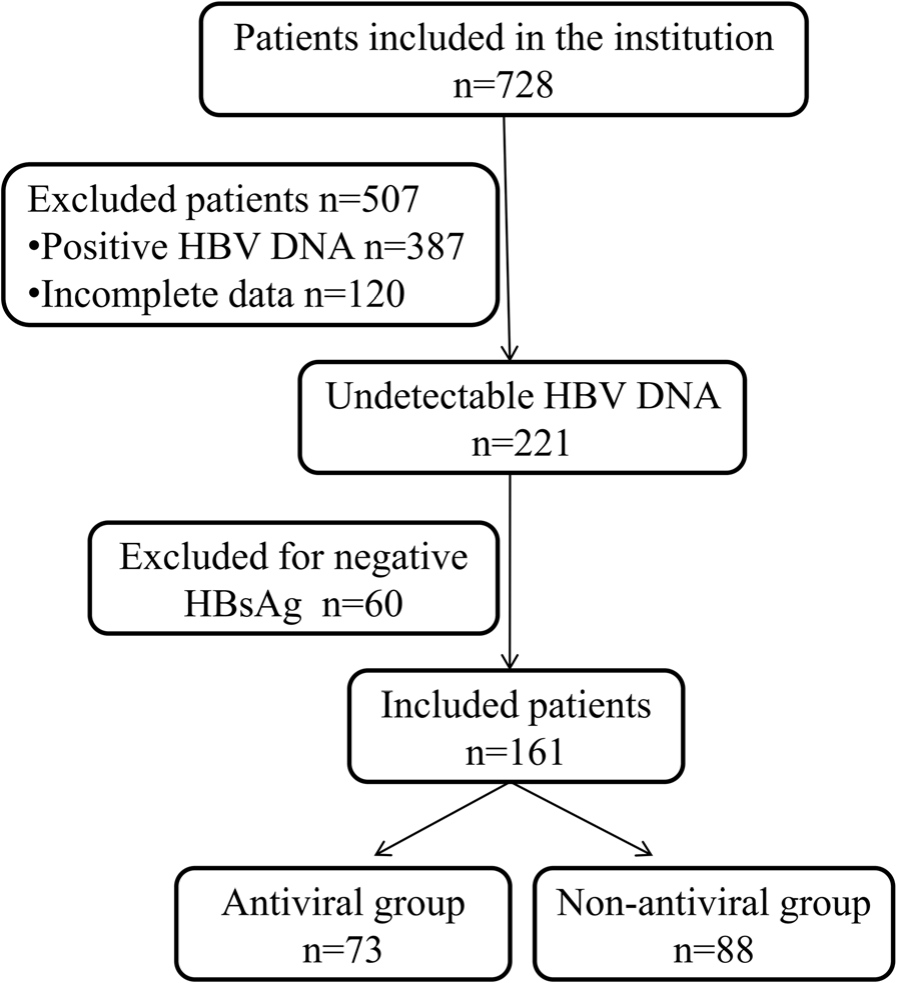
Flowchart for the selection of eligible patients

There were 728 such patients registered in our system. According to the inclusion and exclusion criteria, 161 HCC patients who tested positive for serum HBsAg but negative for HBV DNA before surgery were included. Among the selected 161 patients, 73 (45.34%) received AVT during the perioperative period and were included in the AVT group, while the remaining 88 (54.66%) were included in the non-AVT group (Fig. 1, Additional file 1). All patients were negative for hepatitis C virus (HCV) antibodies; did not receive adjuvant treatments, such as systemic chemotherapy, postoperative adjuvant TACE or transcatheter arterial embolization (TAE), and adoptive immunotherapy; had only HCC; and had the diagnosis of HCC pathologically confirmed after the surgery. During the postoperative follow-up period, patients who experienced HBV reactivation were administered entecavir and still be included in their initial groups.

Before the PSM analysis, most factors were balanced except Barcelona clinic liver cancer (BCLC) stage, tumor size, tumor capsule, vascular invasion, liver cirrhosis, anatomical hepatectomy, and HBV reactivation (all *p* < 0.05; Table 1). After PSM, all measured baseline variables were balanced between AVT group and non-AVT group, and finally, we generated 37 pairs of patients who had no significant differences in terms of all confounding factors for subsequent analysis (Table 1).

**Table 1:** Clinicopathological variables between the two groups

Variables	Before PSM			After PSM		
	Non-antiviral *n* = 88	Antiviral *n* = 73	*P* value	Non-antiviral *n* = 37	Antiviral *n* = 37	*P* value
Gender, M/F	76/12	66/7	0.428	31/6	32/5	0.744
Age (year), < 50/≥ 50	39/49	43/30	0.065	19/18	20/17	0.816
AFP level (ng/ml), < 400/≥ 400	58/30	48/25	0.751	22/15	22/15	1.000
BCLC stage, 0,A/B,C	49/39	55/18	0.009	28/9	24/13	0.309
Tumor size (cm), < 5/≥ 5	40/48	52/21	0.001	26/11	25/12	0.802
Tumor number, < 3/≥ 3	84/4	70/3	0.893	35/2	36/1	0.556
Capsule integrity, yes/no	54/34	62/11	0.001	27/10	26/11	0.797
Satellite nodule, yes/no	7/81	7/66	0.714	5/32	5/32	1.000
Vascular invasion, yes/no	36/52	17/56	0.018	8/29	12/25	0.295
MVI, yes/no	3/85	5/68	0.317	1/36	2/35	0.556
Necrosis, yes/no	18/70	9/64	0.169	4/33	4/33	1.000
Liver cirrhosis, yes/no	41/47	52/21	0.002	19/18	22/15	0.483
PHT, yes/no	32/56	31/42	0.430	13/24	15/22	0.632
Anatomical hepatectomy, yes/no	32/56	39/34	0.030	14/23	15/22	0.812
Blood loss (ml), < 500/≥ 500	75/13	62/11	0.958	31/6	33/4	0.496
Operative time (min), < 120/≥ 120	3/85	7/66	0.106	1/36	0/37	0.314
ALT (U/L), < 40/≥ 40	51/37	44/29	0.766	22/15	23/14	0.812
ALB (g/L), ≤36/> 36	16/72	10/63	0.442	7/30	6/31	0.760
PT (s), < 13/≥ 13	63/25	52/21	0.960	27/10	27/10	1.000
HBV reactivation, yes/no	20/68	2/71	0.001	3/34	2/35	0.643

*PSM* propensity score matching, *AFP* alpha-fetoprotein, *BCLC* Barcelona clinic liver cancer, *MVI* microvascular invasion, *PHT* portal hypertension, *ALT* alanine aminotransferase, *ALB* albumin, *PT* prothrombin time, *HBV* hepatitis B virus

### HBV reactivation

Before PSM, there were two patients (2.74%) presented with HBV reactivation in the AVT group, while 20 patients (22.73%) presented with HBV reactivation in the non-AVT group, and this difference was statistically significant (*p* < 0.01). Patients who experienced reactivation were considered for subsequent antiviral therapy.

### Effect of antiviral therapy on recovery of liver function

**Fig. 2:**
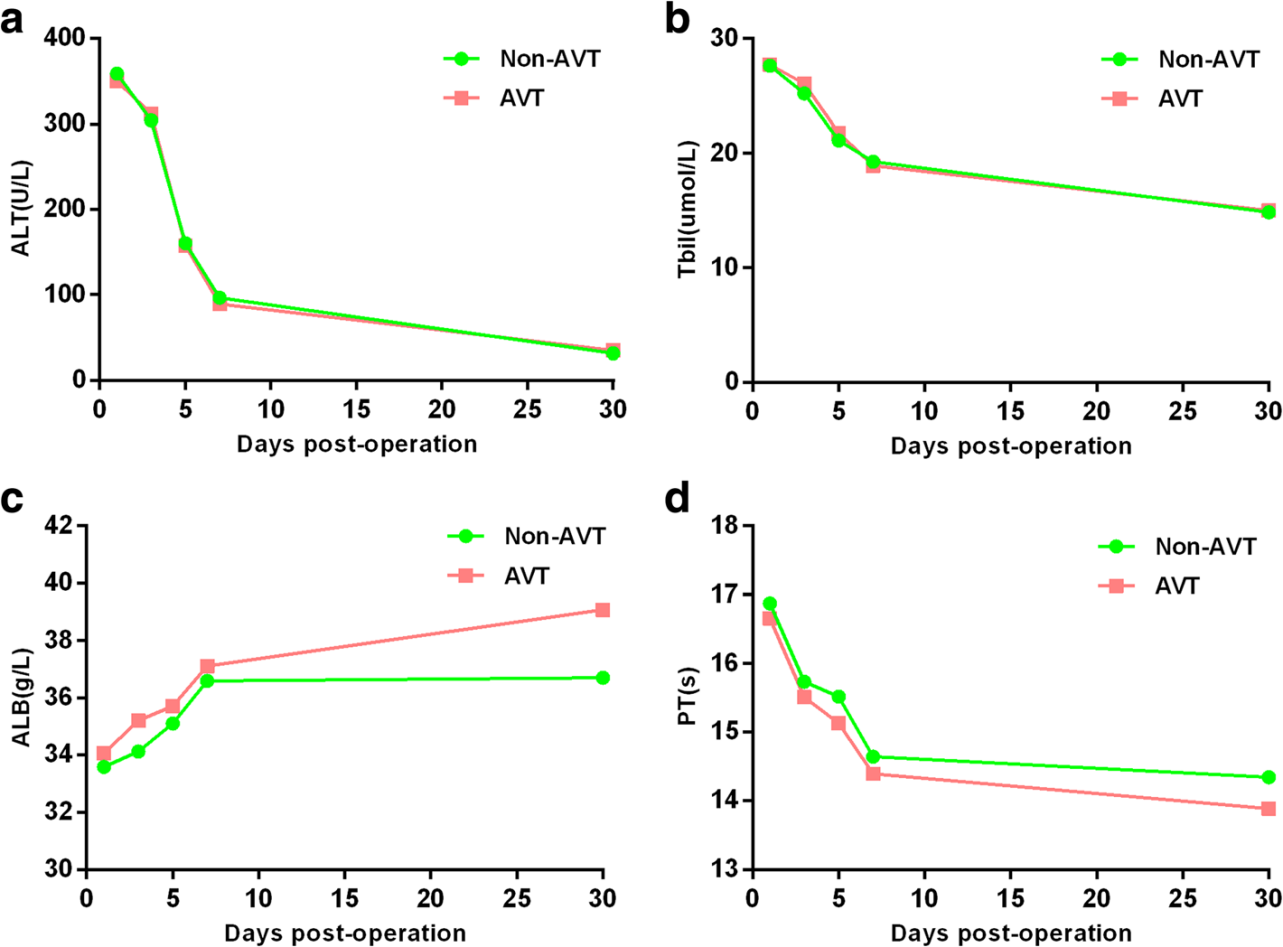
Comparison of liver function between patients with or without perioperative antiviral therapy. ALT alanine aminotransferase, Tbil total bilirubin, ALB albumin, PT prothrombin time

At 1 day, 3 days, 5 days, and 7 days after hepatectomy, liver function parameters such as alanine aminotransferase, total bilirubin, albumin, and prothrombin time all showed no significant difference between AVT and non-AVT groups (Fig. 2a, b, d, all *p* > 0.05). However, albumin was significantly higher in the antiviral group than in the non-antiviral group at 1 month after hepatectomy, indicating better recovery of liver function to some extent (Fig. 2c, *p* < 0.05).

### Independent risk factors of prognosis

The univariate and multivariate analyses are summarized in Table 2. On univariate analysis, BCLC stage, tumor size, tumor number, satellite nodule, blood loss, ALT, and HBV reactivation were identified as significant factors of RFS. Multivariate analysis revealed that tumor number (hazard ratio (HR) 5.39; 95% CI 2.276–12.766; *p* < 0.01) and HBV reactivation (HR 8.037; 95% CI 4.646–13.906; *p* < 0.01) were associated with RFS. Similarly, based on multivariate analysis, tumor size, anatomical hepatectomy, operative time, and HBV reactivation were related to overall survival (Table 3).

**Table 2:** Prognostic factors associated with recurrence-free survival

	Univariate analysis			Multivariate analysis		
	*p* value	HR	95% CI	*p* value	HR	95% CI
Gender	0.259					
Age	0.895					
AFP	0.381					
BCLC	0.044	1.656	1.013–2.707			
Tumor size	0.040	1.663	1.022–2.706			
Tumor number	0.001	4.288	1.843–9.978	**<** 0.01	5.390	2.276–12.766
Capsule integrity	0.650					
Satellite nodule	0.018	2.352	1.161–4.763			
Vascular invasion	0.215					
MVI	0.582					
Necrosis	0.199					
Liver cirrhosis	0.077					
PHT	0.636					
Anatomical hepatectomy	0.137					
Blood loss	0.028	1.946	1.076–3.517			
Operative time	0.509					
ALT	0.042	1.655	1.017–2.693			
ALB	0.726					
TB	0.130					
PT	0.757					
Antiviral therapy	0.280					
HBV reactivation	**<** 0.01	7.490	4.359–12.870	**<** 0.01	8.037	4.646–13.906

*AFP* alpha-fetoprotein, *BCLC* Barcelona clinic liver cancer, *MVI* microvascular invasion, *PHT* portal hypertension, *ALT* alanine aminotransferase, *ALB* albumin, *TB* total bilirubin, *PT* prothrombin time, *HBV* hepatitis B virus

**Table 3:** Prognostic factors associated with overall survival

	Univariate analysis			Multivariate analysis		
	*p* value	HR	95% CI	*p* value	HR	95% CI
Gender	0.680					
Age	0.925					
AFP	0.403					
BCLC	0.018	2.320	1.158–4.645			
Tumor size	0.001	3.875	1.793–8.376	< 0.01	5.092	2.174–11.926
Tumor number	0.066					
Capsule integrity	0.920					
Satellite nodule	0.811					
Vascular invasion	0.017	2.319	1.160–4.639			
MVI	0.634					
Necrosis	0.351					
Liver cirrhosis	0.820					
PHT	0.365					
Anatomical hepatectomy	0.044	0.484	0.239–0.981	0.034	0.463	0.227–0.944
Blood loss	0.051					
Operative time	0.009	3.590	1.379–9.345	< 0.01	7.064	2.433–20.512
ALT	0.986					
ALB	0.654					
TB	0.214					
PT	0.629					
Antiviral therapy	0.016	0.375	0.168–0.834			
HBV reactivation	< 0.01	5.353	2.641–10.848	< 0.01	5.190	2.545–10.582

*AFP* alpha-fetoprotein, *BCLC* Barcelona clinic liver cancer, *MVI* microvascular invasion, *PHT* portal hypertension, *ALT* alanine aminotransferase, *ALB* albumin, *TB* total bilirubin, *PT* prothrombin time, *HBV* hepatitis B virus

### Survival analysis before and after propensity matching analysis

**Fig. 3:**
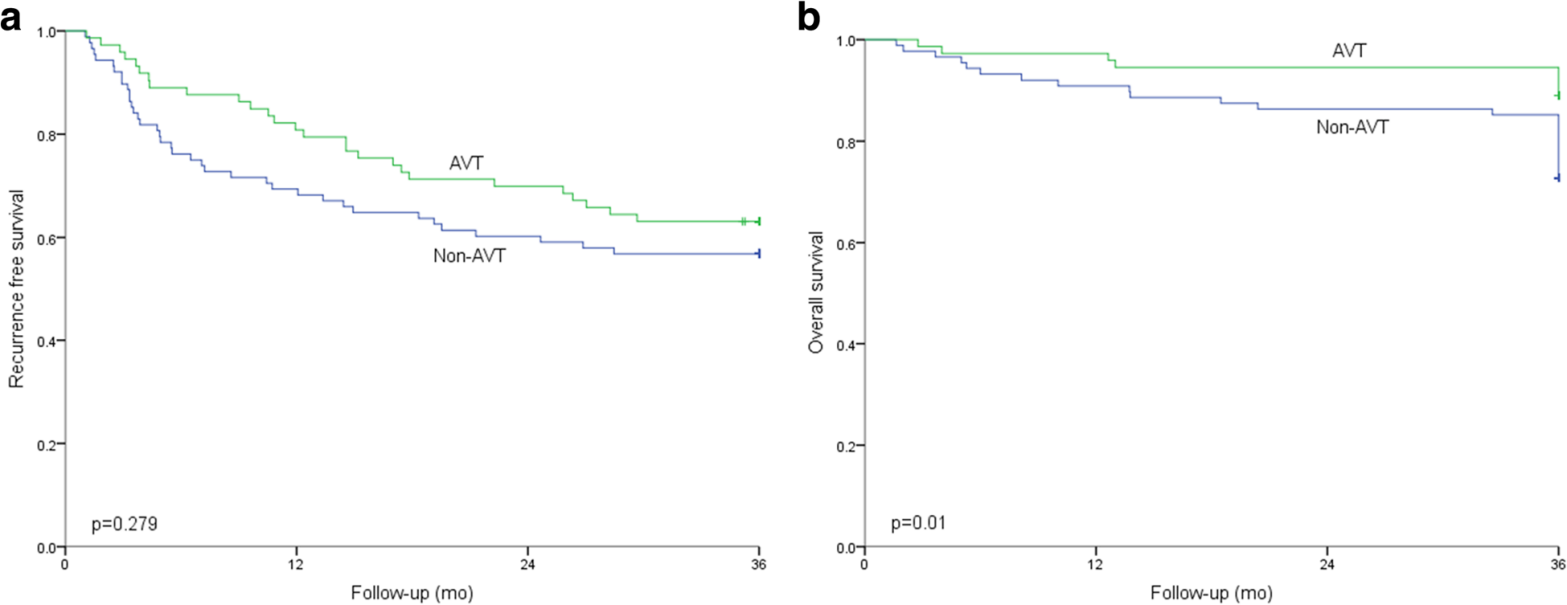
Comparison of RFS and OS between the two groups before PSM

**Fig. 4:**
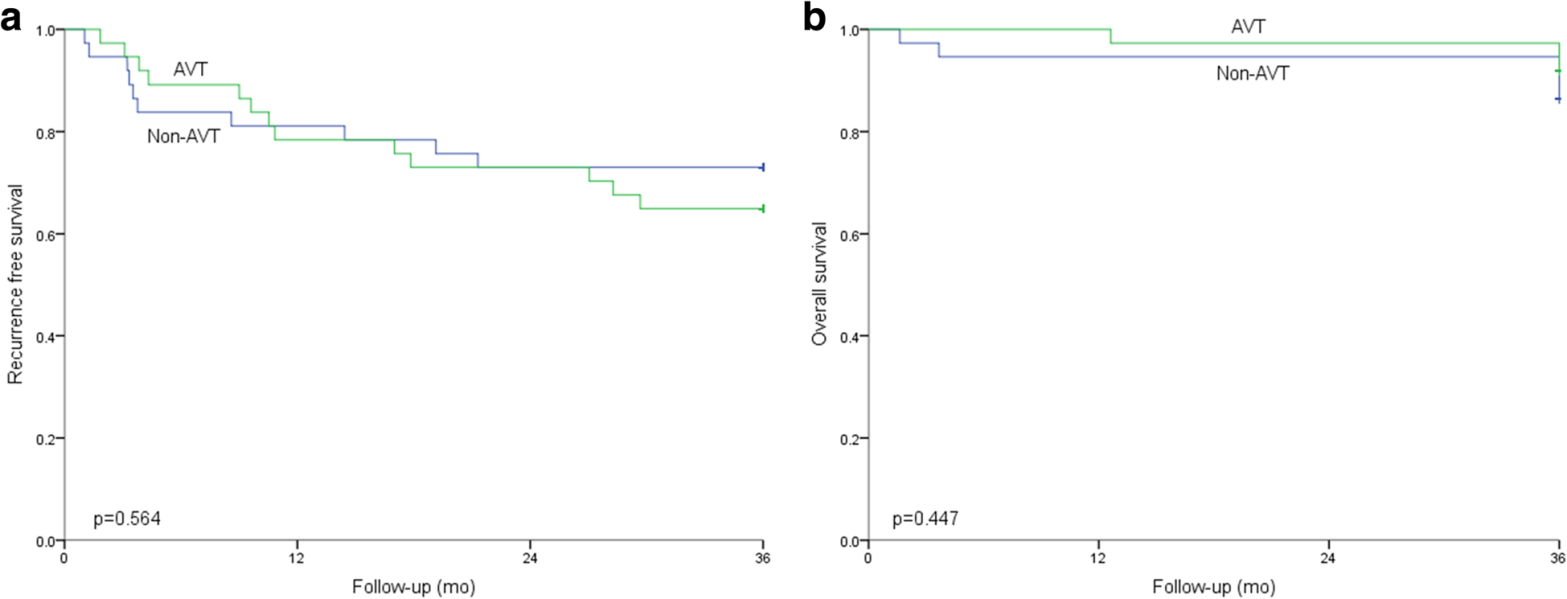
Comparison of RFS and OS between the two groups after PSM

Before propensity matching analysis, the 1-, 2-, and 3-year RFS rates were 80.82%, 69.86%, and 58.90% and 69.32%, 60.23%, and 55.68% in the AVT group and control group, respectively (Fig. 3a, *p* = 0.279); the 1-, 2-, and 3-year OS rates were 97.26%, 94.52%, and 93.15% and 90.91%, 86.36%, and 84.09% in the AVT group and non-AVT group, respectively (Fig. 3b, *p* < 0.05). After PSM, the 1-, 2-, and 3-year RFS rates in the AVT group and the non-AVT group were 78.38%, 72.97%, and 62.16% and 81.08%, 72.97%, and 72.97%, respectively (Fig. 4a, *p* = 0.564); however, the 1-, 2-, and 3-year OS rates were 97.30%, 97.3%, and 91.89% and 94.59%, 94.59%, and 86.49%, respectively, and there was no significant difference between the two groups (Fig. 4b, *p* = 0.447).

## Discussion

For patients who are positive for HBV DNA, official guidelines recommend antiviral therapy and rational drug selection [25–27]. Previous studies have indicated the necessity of antiviral treatment for HCC patients with chronic HBV infection; however, it remains controversial whether it is also necessary to implement antiviral treatment for patients with undetectable levels of HBV DNA [28, 29]. Similarly, HCC patients who are negative for HBV DNA also encounter the uncertainty.

The high recurrence rate and the development of postoperative metastasis are always important issues for clinicians and patients. It has been reported that the HCC recurrence rate within 5 years after hepatectomy is as high as 70% [30–32]. Tumor size, tumor number, tumor stage, tumor differentiation, MVI, etc. are all factors that influence postoperative recurrence in HCC patients [33, 34], and this is also consistent with results of our univariate and multivariate analyses (Table 2). At the same time, it is clear that a positive HBV DNA test is an independent risk factor for postoperative recurrence. Numerous studies have shown that a high serum level of HBV DNA (more than 2000 IU/ml) is an independent risk factor for the development of HCC in chronic hepatitis B patients, and higher serum levels of HBV DNA are more likely to be associated with the occurrence of liver cancer than lower levels of HBV DNA [35–37]. It has also been confirmed that surgery may cause HBV reactivation with a high viral load, thus increasing the risk of tumor recurrence; therefore, AVT is required for those patients [15, 38]. However, studies about patients with very low viral load levels, such as the HBV DNA level < 100 IU/ml used in this study, are almost nonexistent, and currently, there are no guidelines for those patients.

To the best of our knowledge, this is the first study exploring the effects of AVT on prognoses in HCC patients who are positive for serum HBsAg but negative for HBV DNA (HBV DNA < 100 IU/ml).

Our results showed that antiviral therapy can substantially reduce the postoperative HBV reactivation rate, which was consistent with the results of previous studies [39–41]. In terms of HBV reactivation, there is no exact mechanism that has been proposed to explain the mechanism by which HBV reactivation occurs after surgery. Some scholars believe that the surgery itself leads to this outcome because hepatectomy may induce immune-suppression [42–44]. Other studies suggest that the presence of covalently closed circular DNA in hepatocyte nuclei may be the reason for later reactivation [45–47]. Taken together, the administration of perioperative AVT for a prolonged period of time, such as the 1 year used in this study, perhaps would be helpful to reduce HBV reactivation.

Our results indicate that perioperative antiviral therapy does not improve liver function in 1 week after hepatectomy, but can better promote albumin recovery than non-antiviral therapy at 1 month, which is consistent with previous studies [15, 48]. The reason for the results, we suppose, is the temporality of antiviral therapy, that is, when early stage after operation, the hepatectomy itself dominates the recovery of liver function; however, with the regeneration of residual liver, the influence of surgical factor gradually weakens and the influence of antiviral therapy gradually strengthens.

In this study, we concluded that antiviral therapy may not improve RFS. On the other hand, although there was no significant difference in OS rates between the two groups, we still observed that OS in the antiviral group was higher than that in the non-antiviral group; we hypothesized that the lack of a significant difference may be the result of the small sample size, and studies with larger samples sizes are needed in the future. The conclusion of OS rates was different from that of a previous study [49], and the main reason for this difference may have been the different HBV DNA levels between the two studies. To define a patient as having a negative result for HBV DNA, we had a more stringent standard (HBV DNA < 100 IU/ml), which was much lower than the standard used in the other study (HBV DNA < 1.0 × 10^3^ IU/ml). Perhaps, with such an extremely low viral load, HBV was in a very stable state and seldom caused inflammation of the liver or an immune response; thus, AVT was not be able to exert any influence on tumor recurrence and patient survival. Of course, this conjecture requires more supporting research.

This study had some limitations. First, it was a single-center study. Second, the research period was large, and the importance of AVT for HCC patients has gradually changed over the years, leading to the large initial baseline heterogeneity of patients administered AVT. Third, given that this study was a retrospective study, the duration of AVT was inconsistent. In addition, the sample size may have been a limiting factor for the separation of the KM curves when analyzing OS, and the role played by the tumor-immune microenvironment during this process is still unknown. At the same time, patients’ data about HBeAg, anti-HBe, and HBV phase was absent restricted by the detection means.

## Conclusions

In conclusion, AVT can reduce the HBV reactivation rate but has no effect on survival for HCC patients who are negative for HBV DNA (HBV DNA < 100 IU/ml). This conclusion will be useful for selecting treatment methods for this type of patient. However, studies with larger sample size are needed to further verify this conclusion and explore the possible mechanism underlying this result.

## Additional files

Additional file 1: Eligible patients included in the AVT and non-AVT group. (XLSX 68 kb)

## Abbreviations

AFP: Alpha-fetoprotein
ALB: Albumin
ALT: Alanine aminotransferase
AVT: Antiviral therapy
BCLC: Barcelona clinic liver cancer
CT: Computed tomography
HBV: Hepatitis B virus
HCC: Hepatocellular carcinoma
HCV: Hepatitis C virus
HVI: Hepatic vein invasion
MRI: Magnetic resonance imaging
MVI: Microvascular invasion
OS: Overall survival
PHT: Portal hypertension
PSM: Propensity score matching
PT: Prothrombin time
PVI: Portal vein invasion
RFS: Recurrence-free survival
TACE: Transcatheter arterial chemoembolization
TAE: Transcatheter arterial embolization
TB: Total bilirubin
VR: Virological response
